# Facebook reactions in the context of politics and social issues: a systematic literature review

**DOI:** 10.3389/fsoc.2024.1379265

**Published:** 2024-05-15

**Authors:** Sawood Anwar, Fabio Giglietto

**Affiliations:** Department of Communication and Humanities, University of Urbino Carlo Bo, Urbino, Italy

**Keywords:** Facebook, reactions, emotion, emoji, politics, social issue

## Abstract

In February 2016, Facebook expanded the original “Like” button by introducing five additional “Reactions”—Love, Haha, Wow, Sad, and Angry—using modified versions of Unicode emojis. These reactions enable users to express more nuanced emotions towards posts. This literature review investigates scholarly research on user behavior in response to these reactions, with a focus on a broad spectrum of socioeconomic and psychological issues. We conducted a systematic search across databases including Scopus and Google Scholar, using keywords such as “Facebook” and “Reaction,” combined with various key phrases and Boolean operators. Our review synthesizes sixty-four articles published from 2016 to 2023, exploring diverse topics such as political news, far-right and extremist parties, racism, and hate speech during the COVID-19 pandemic. We organized these articles by theme and publication date. Our meta-analysis reveals that lifestyle and entertainment posts predominantly receive positive reactions, while sociopolitical content tends to elicit a broader spectrum of emotions, including negative sentiments. Furthermore, emotionally charged content consistently attracts higher volumes of reactions, regardless of sentiment. This research highlights the intricate relationship between user reactions and content characteristics, providing deeper insights into the dynamics of online engagement. By understanding these interaction patterns, we gain a better grasp of emotional responses and engagement levels, which ultimately shape online discourse and user interactions.

## 1 Introduction

Social media platforms provide information and communication among a diverse population in terms of different cultural backgrounds and identities with different opinions (Franz et al., 2019). Facebook has become one of the most dominant social networking sites (SNS), and its rise is an important trend, over the past decade (Caers et al., 2013). The number of Facebook users is growing by 1.3% year-over-year, which amounts to an increase of 39 million users (Newberry, 2023). In “Meta Reports Third Quarter 2023 Results,” Facebook announced that it had 2.09 billion daily active users on average for September 2023, an increase of 5% year-over-year, while Facebook monthly active users were 3.05 billion as of September 30, 2023, an increase of 3% year-over-year (Menlo Park and California, 2023).

Since Facebook’s launch on February 4, 2004 (Onion et al., 2019), its ongoing evolution process has been an interplay between “platformisation” and “infra-structuralisation”; the Meta (Previously known as Facebook) is subjected to continuous change (Helmond et al., 2019).

In 2009, Facebook added the “Like” button, which was, in 2010, added to individual comments to posts (Eranti and Lonkila, 2015). This button and another kind of lightweight affirmation, that is, “Facebook reactions,” served as social cues of acceptance and for interpersonal relationships (Scissors et al., 2016). All linguistic and non-verbal communicative channels are open to interpretation, which can be phatic where the “Like” button facilitates high ambiguity and seems to have a multipurpose reaction for any content worthy of social response.

In 2016, Facebook extended this functionary button to a single positive of “Like” and allowed users to react to SNS content using a limited range of emotional reactions such as “love,” “haha,” “wow,” “sad,” and “angry”; this function was allowed to expand to comment in 2017 (Jalan, 2023). They represent a light version of Unicode emojis indicated by an icon resembling common graphic substitutions for ASCII emoticons (Paolillo, 2023), which allow users to express how they feel about the contents.

The emotion underlying these six reactions is considered to be universal and frequent (Tian et al., 2017) and to convey emotive meaning, including feelings, and moods specific to posts and contents. A Facebook reaction contains less text-based information than comments on a post or writing on the wall, though it is still over-generated content and has valued information at first sight. In particular, as Facebook posts receive more reactions, it becomes more visible and noticeable to other users.

The area of Paralinguistic digital affordance (PDA) is a related light form of non-verbal communication where the response to another person’s social media content (Hayes et al., 2016), is used to convey the message and relation-based sentiment where the user clicks a single button and others interpret the meaning (Sumner et al., 2020); thus, Facebook reactions have the nature of PDA.

The newly introduced “Facebook reactions” can better clarify the user’s intended meaning, which would not be possible with a single one-click “Like” button; these Reaction buttons are fairly aligned with the desired button where users can focus on the content-based meaning in emotional ways. Facebook reactions can be defined as various non-verbal cues that might save time by speeding up the rate at which communicators can share information and develop relationships (Sumner and Ramirez, 2017).

If we consider the impact of the Reaction on overall engagement by characterizing different engagement behaviors on two dimensions (i.e., the level of cognitive mode and effort required and the emotional state of expression), reactions—the new feature—inherit the low cognitive effort of “Like” that supports the expressions of major emotional complexity (Al-Rawi, 2019; Sumner et al., 2020); thus, scholars used reactions to investigate user behavior and situation-based current topics to measure various aspect of socioeconomic and psychological issues (Kosinski et al., 2015; Godard and Holtzman, 2022), concerning political issues, elections, public debate, health issues, and the trustworthiness of the news about current topics or events. Thus, Facebook provides an environment where researchers can investigate public reactions in a relatively convenient way (Kim and Yang, 2017).

The primary topics were either related to the introduction of “Reactions and their impact” (Hutchinson, 2016; Turnbull and Jenkins, 2016), “the linguistics approach” or “Emotion detection” (Tian et al., 2017), which seemed to dawn a new era of “Facebook and the user’s engagement” through Facebook reactions. However, we have observed that the topics covering political issues (Tasente and Rus, 2019), and others related to human behavior, health, and commerce (Antoniadis et al., 2019) are getting diversified. There are many articles related to overlapping topics. However, it is difficult to identify or maintain a particular theme, although the results cover two or more identical results with comparative analysis, and it is possible to put one article into more than one categorized topic.

This literature review focuses on “Facebook reactions,” examines the literature, and serves as a comprehensive and cumulative approach, summarizing the existing studies and research articles scattered under different subjects and scientific fields.

However, it also aims to get comparable results within broader classifications and themes. The main objective of this review is to offer a consolidated and comprehensive perspective to identify the common thread and overarching themes within the literature. By synthesizing all the findings from numerous studies, the review would shed light on the role and impact of Facebook reactions.

Thus, we seek how “Facebook reactions” play a role in different or comparable topics and their results. There are other open questions related to Facebook reactions, such as the importance of Facebook reactions for the users and correspondence of posts. What aspects of posts or topics are meaningful? Do different topics acquire different reactions and to what extent?

## 2 Methodology

### 2.1 Selection procedure

The focus of the literature review is limited to Facebook. This decision is appropriate because, at present, Facebook is used worldwide and is the most popular SNS (Noyes, 2022; Newberry, 2023). Moreover, the emphasis is based on empirical studies and their findings rather than non-empirical studies. We adhere to the Five-step process of systematic review outlined by Briner and Denyer (2012): (1) formulate the research questions, (2) find studies, (3) select and evaluate the studies, (4) analyze the findings, and (5) report the results.

**Figure 1:**
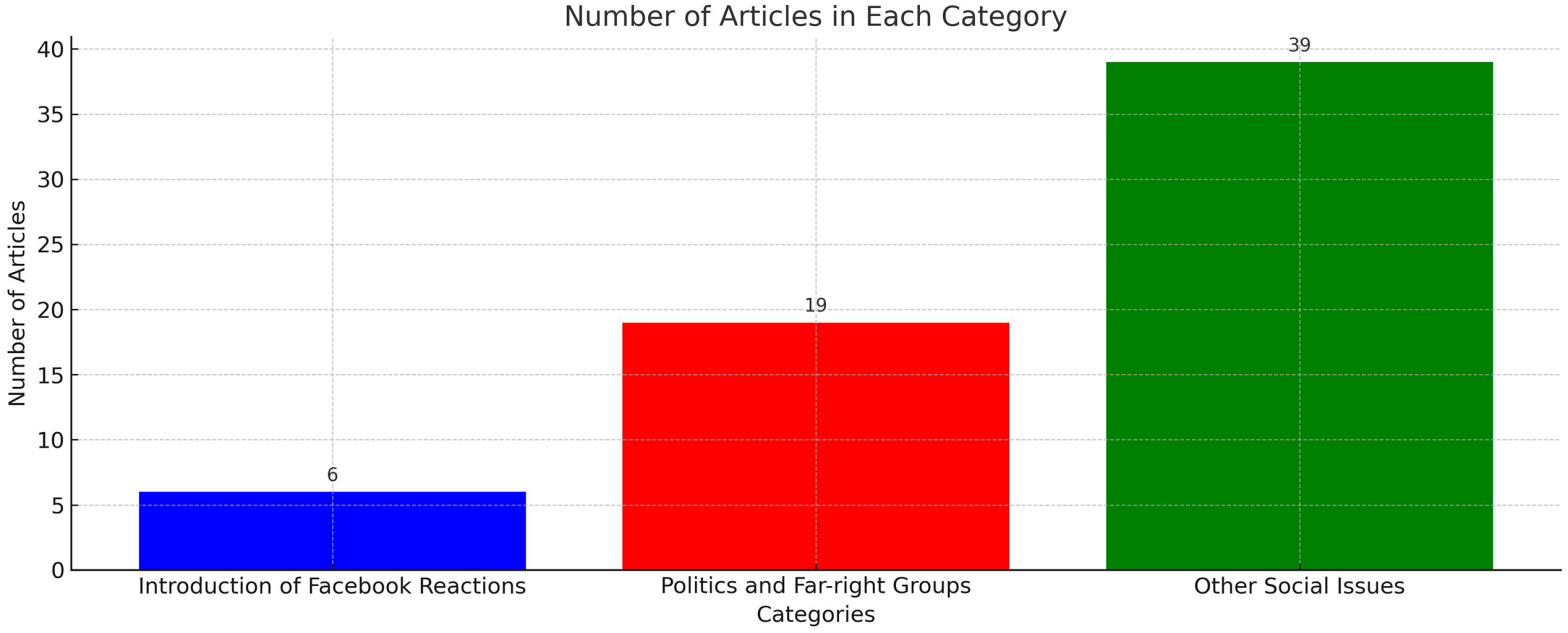
A total of 64 published articles analyzed between 2016 and 2023.

Articles were collected from Scopus, Google Scholar, and other search engines. We used the keywords “Facebook” and “Reaction,” with a combination of different key phrases and Boolean operators. In this review, the selected articles are either published in peer-reviewed scientific journals or conferences before proceeding through the same process. The search was performed every month from July to September 2023. It returned a total of 64 articles published between 2016 and 2023. Matrix and Reference lists were read and scanned with different topics classified across the entire publication time frame (Figure 1).

#### 2.1.1 Data analysis

We followed the guidelines marked by Creswell (2009) who states that a review should summarize the cumulative knowledge based on the topic and highlight the issues that research has yet to solve (Table 1).

**Table 1:** List of major Facebook research topics.

Topic	Sub-topic
(A) Introduction of Facebook reactions	Introduction to Facebook, language and linguistics.
(B) Politics and far-right groups	(a) Political news, (b) Far-right and extremist parties, racism, hate speech with COVID-19.
(C) Other social issues	Psychological, sentimental, and emotional; health care, business and customer engagement, miscellaneous.

The data analysis procedure is as follows. Each empirical article was read and summarized through their research questions including their results and conclusions. Using the comparative naturalistic method (Armstrong, 2010), we read each article and noted the content to examine its tentative research topic. The finding of the article was also noted. The same procedure was repeated with succeeding articles, regardless of whether they were similar to the previous article. If there was any similarity, we placed the article under the first categorized research topic and then went through another article.

We repeated this process for all the articles, although it was possible to categorize one article into more than one research topic.

#### 2.1.2 Topic categorization with results

To provide correspondence between the topic and their content including their result output, we have categorized a wide range of topics/information labeled as (1) Introduction to Facebook reactions, (2) Language & Linguistic, (3) Psychological, Sentimental, and Emotional, (4) (a) Political News, (4) (b) Far-right and Extremist Parties, Racism, Hate speech with COVID-19, (5) HealthCare, (6) Business and Customer engagement, and (7) Miscellaneous.

Identified research categorizations appeared to cluster into the major groups – (A) Introduction of Facebook reactions, (B) Politics and Far-right groups, and (C) Other social issues. Please find below the table of major groups and topic categorization.

## 3 Analysis

### 3.1 Overview of the article published over the timeframe analyzed

Since Facebook launched its Reaction button, researchers have put their efforts into this new feature. In the beginning, the articles were related to Facebook reactions and their usage, their impact, and their introduction to user engagement. Later, the coverage area of those articles became vast and spread over every field of social issues such as political communication to Psychological, Sentimental, Emotional, Health Care, Business and Customer engagement, and so on Figure 1.

The categories are Introduction of Facebook reactions (06 Articles), Politics and Far-right Groups (19 Articles), and Other Social Issues (39 Articles) (Supplementary material).

Given those categories, in the next section, we describe the type of scientific work and the main findings identified in each category.

#### 3.1.1 Introduction of Facebook reactions

Facebook launched “Reaction” buttons globally on February 24, 2016 (Krug, 2016). Facebook named these emojis as love reaction (heart emoji), haha reaction (laughing face), wow reaction (surprised facial expression), sad reaction (sad face), and angry reaction (angry face) (Kluknavská et al., 2023).

Since then, many articles have been published, which have primarily explored the new features and users’ engagement with them. Researchers have been considering and putting their perspectives and opinions on “Facebook Reactions” and “their usage in upcoming days”; for example, Turnbull and Jenkins (2016) examined Facebook’s evolution process, and how this new feature provided an opportunity to gain a better understanding of how users engage emotionally. Wisniewski et al. (2020) examined users’ feedback about newly introduced features, design implications, and the signification of “Facebook reactions”; The authors argue that, after the launch, users were more positive about the feature of Facebook reactions, and their misconceptions were clarified with actual use. However, there are many constraints including users’ inability to express emotions or conflict of emotions.

In the early years, we found articles measuring “Facebook reactions” from behavioral responses to evaluating emotional responses.

Varanasi et al. (2018) argue that “Facebook reactions” help us to understand relationship maintenance and group cohesion; thus, the new feature has user benefits. Moreover, they found a direct effect of “Facebook reactions” as “bridging and bonding to social capital” (Coleman, 1988) and a significant relationship between receiving reactions and bridging social capital. This study gives a clear understanding of SNS features, that is, “Facebook reactions” to the sociopsychological elements of users.

Smoliarova et al. (2018) address the usage of “Facebook reactions” on social media that correlate with different types of Facebook reactions, sharing, and commenting activities. They discussed the possible meaning of the homogeneity of emotions shared on the Facebook community page. Furthermore, the study provides a new pattern and a new insight into user engagement with content on Facebook with its Reaction features.

Merrill and Oremus (2021) have found that emojis (Facebook reactions) are five times more valuable than “Like,” and “reactions” are used as a signal to push more emotionally provocative content. Based on the theory that “Posts that prompted lots of reaction emoji tended to keep users more engaged, and keeping users engaged was the key to Facebook’s businesses,” they debated over the “anger” reaction and identified its effect and human judgments that underlie Facebook algorithm.

PDA is a light form of non-verbal communication provided in response to another person’s social media content (Hayes et al., 2016); it is used to convey the content message and relation-based sentiment where users can click a single button and let others interpret their meaning (Sumner et al., 2020); thus, “Facebook reactions” allows to explore the nature of PDA.

Sumner et al. (2020) have found that the social media platforms that offer multiple one-click response cues may afford different communicative opportunities than those who have a single PDA response opinion such as “Like” (perceived more faithfully than Reactions); “Like” and “Love” cues were most faithful, whereas other reactions were perceived more deliberate and less automatic communicative behaviors than “Like.” Carr et al. (2018) presented explanatory mechanisms behind the use of PDA responses, using the social comparison theory, social penetration theory, and expectancy violations theory for a successful post. They have found that social comparison and communicative reciprocity provide the exploratory power to “Like” and “Reaction” for a successful Post.

On the contrary, Paolillo (2023) found that the reaction distribution across his sample was complex, unstable, and did not reveal a face-work pattern; thus, caution is important for interpreting quantitative analyses for the feature of “Facebook reactions.”

Other than English, much research is ongoing to explore the dimensions of Facebook reactions in different languages. Using the Indo-Aryan language Sinhala or Sinhalese used in Sri Lanka (Fernando, 1977), Weeraprameshwara et al. (2022) created a dataset using “‘Facebook reactions” and explored the effectiveness of one-tiered and two-tiered embedding architectures in Sinhala text. In another study for Sinhala Posts, Jayawickrama et al. (2022) considered the Baseline Model to predict Facebook reactions and used the phrase “embedded architecture” as well. Rahman and Seddiqui (2019) experimented with emotional analysis with the text corpus in the Bangla language consisting of user comments with Facebook reactions.

### 3.2 Politics and far-right groups

Researchers have explored the influence of Facebook and its reaction to political motivation since Facebook launched its Reaction feature globally. In the beginning, Turnbull and Jenkins (2016) predicted that “Facebook reactions” would be used in different directions or areas as they argued that this feature would be good and useful for evaluating social media campaigns including business purposes and considered a parameter for emotional objects for communication.

Notably, “Facebook reactions” is the diversion or expansion of the previous functional approach of the “Like” button (Jalan, 2023). Larsson (2017) presented his analysis of “Facebook reactions” as the diversification of “Like” for commenting and sharing on the Newspaper Facebook pages. The feature emerged as unpopular compared to the original “Like” functional button, and both negative and positive reactions were used on the newspaper posts. Presumably, users were not too familiar with the usage of Facebook reactions. Bagić Babac (2022) attempted to establish a set of rules by connecting different emotions using “Facebook reactions” for news posts and comments and measuring positive and negative emotions encoded in the content. She reports that emotion acts as a stimulus to a particular pattern of behavior; positive and negative emotions usually influence positive and negative comments, respectively. Pérez-Seoane et al. (2023) analyzed the publications with the highest volume of interactions and found emotional reactions associated with positive feelings.

#### 3.2.1 “Facebook reactions” in political campaigns and elections

Subsequently, studies show that politicians are also using social media, especially Facebook to explore its deliberative potential, and their digital followership influences the number of “Facebook reactions” (Keller and Kleinen-von Königslöw, 2018).

That result was proven in the local election of Mexico where political parties had a large impact with few posts although the candidates had terrible public perception. “Facebook reactions” opened a path to perceive voter’s interest and became a new communication channel, al though the election results could not be predicted (Sandoval-Almazan and Valle-Cruz, 2018). A similar study conducted by Sandoval-Almazan and Valle-Cruz (2020) aims to answer the question of whether the “Facebook reactions” in Mexico reflect the outcomes of the election in June 2017 and what possible users’ emotions are generated with regards to political candidates. That investigation assesses sentiment as an indicator of the mood of public opinion, which could be expressed through “Facebook reactions” in political campaigns.

The finding shows that the winning political party got more negative sentiment, and the parties that got the highest positive sentiment and more reactions over their posts did not win the election. It shows that emotions expressed through reactions are a way of assessing voter behavior that is not easily predicted.

In the Mexican elections, de León and Trilling (2021) conducted a study focusing on positive versus negative political content related to emotional responses, and the degree of influence of the articles that were shared across social media in the context of that election. They found a negative bias in news-sharing behavior and engagement and a great amount of “anger” toward political news. Using a strong dominance of “anger” reaction in that political news during the election, they found that the election was marked with violence and corruption.

Bil-Jaruzelska and Monzer (2022) examined the relationship between emotions and user engagement with political Facebook posts during the Brexit referendum, and found that engagement with a post substantially increased when appealing to anger, enthusiasm, and pride. A relationship between appeals to specific emotions (i.e., fear, anger, enthusiasm, and pride) and the engagement of users was observed.

Moreover, with regards to the relationship between topics, emotions, and user engagement, Gerbaudo et al. (2019) analyzed posts and comments through “Facebook reactions” and found that the positivity of topics was more tended and fairer than negative and controversial issues. They also state that negative campaigning and negative emotions do not always tend to prevail on Facebook, although they found a correlation between positive content and user engagement. Klinger et al. (2023) studied negativity, dramatization, and populist content on Facebook during the election campaigns of the European Parliament election (2014–19) across twelve countries. They found that a higher frequency of likes, shares, and comments had a deeper impact on social media platforms and reached more audiences. Facebook algorithms were changed in 2016 during the election campaigning, and the impact could be observed on user engagement before and after the launching of “reactions.” The users had six more responsive options (i.e., like, love, haha, wow, angry, sad), and negative campaigning with posts evoking negative emotions and dramatization yielded more user engagement than other posts. Notably, the populist content also got more user engagement during 2014 before the Reaction button launched.

Furthermore, considering the negative sentiment with user (voter) engagement, Macdonald et al. (2023) have found that negativity is the consistent factor for user (voters) connections on Facebook, where more negative reactions/words generate higher user engagement. According to the authors, candidates strategically employ negativity in their posts to encourage engagement. The increased engagement oftentimes matches the sentiment with the post and negative messages and results in more negative reactions (“sad” and “anger”). A link exists between a tone set by the candidates who appeal emotionally and triggers the user who follows the candidate on pages, resulting in increased engagement.

#### 3.2.2 “Facebook reactions” in populist politics

In the case of Swiss politicians, a large fellowship and their reactions increase the visibility of political messages or actors and result in more media coverage (Keller and Kleinen-von Königslöw, 2018). Their followership is more prone toward populist parties. A comparison between two populist parties in two countries (Italy and the United Kingdom) shows a measure of emotional response, which is employed as “Facebook reactions,” allowing them to indicate their emotional value over a post that provokes them (Mancosu, 2018).

After “Facebook reactions” was launched, user engagement saw an unprecedented increase. Klinger et al. (2023) found that negativity, dramatization, including more populist content engaged more in negative campaigning during the European Parliament election in 2019. After examining how populist leaders use Facebook and if they elicit more emotional reactions, Sandberg et al. (2022) have concluded that populist party leaders use Facebook to reach out to their community prolifically. Their posts activate feelings of indignation and trigger responses that are more emotional. The messages posted by populist leaders receive more reactions than only “Like”; in particular, “haha” and “anger’ are prominent. It is interesting that political communication on Facebook triggers emotional reactions and influences the frequency of post-sharing, reflecting sadness with triggered angry reactions (Zerback and Wirz, 2021).

It was unclear whether the popularity of the populist post was driven by the “nature of the message” or “by populist actors” or through both factors’ interaction. Blassnig and Wirz (2019) have found that both populist messages and actors influence user perception of the post. Moreover, analyses of populism and user reactions show the effect of populist communication on user reaction. Populist leaders always use populist communication strategies to increase the outreach of their messages and then stimulate the users to interact with the message, Jost et al. (2020) studied this phenomenon during the German federal election campaign in 2017; they took “anger” and “love” Facebook reactions into consideration and found exclusive populist message features increase the “anger” reactions, whereas inclusive populism and positive depiction of ordinary citizen lead a high number of “love” reactions and lesser number of “anger” reactions (Jost et al., 2020). Negative emotions such as anger seem to be reflected when people come across negative information.

Similarly, Kluknavská et al. (2023) have found that leaders mobilize followers through carefully crafted messages that appeal to their emotions. Understandably, political parties use specific messages to trigger negative emotions in their followers to incorporate and mobilize them. Klinger et al. (2023) also showed that political parties across the twelve countries during the 2019 European Parliament elections evoked negative emotions and engaged in more negative campaigning by including populist content in their posts. Donald Trump used messages that frequently got various reactions from, “love” to “anger,” “wow” to “sad,” and “haha” to “like”; however, the posts that generated “love” and “sad” reactions received the highest user engagement compared to others (Tasente and Rus, 2019).

#### 3.2.3 “Facebook reactions” in far-right politics

The “Facebook reactions” feature provides opportunities to investigate global emotional responses to political messages on Facebook, which is a common platform. Muraoka et al. (2021) described a new measure and presented a dataset of 690 political parties in 79 democracies. They studied “love” and “anger” reactions to the posts and measured the potential usage of emotional responses, with party-wise variation in the frequency of such reactions. Their results for parties received systematically different proportions of the popular reactions “love” and “anger,” which depended upon the ideology, party line, and populist orientation. The greater emotional responses came from more extremist parties; other nationalist, populist, and right-leaning parties received a relatively higher proportion of “anger” reaction as well as emotional polarization. Negative and volatile categorical reactions such as “anger,” “haha,” and “wow” primarily drive controversy, with a high sharing ratio of controversial news. Oliveira and Azevedo (2023) reveal that negative emotions do not always indicate the presence of hate speech and negativity. These are implications for detecting hate speech.

Considering the example of the Belgian far-right political party, Vlaams Belang, Matamoros Fernandez (2018) noticed that the party used Facebook reaction to spread anger and the audience responded to their call by posting more emojis to express their rage and racist tropes. She explored everyday racism manifested on Facebook as a combination of platform affordances with emojis. Thus, the study of emoji is also related to visual content, hate speech, and content moderation.

It was unclear which features of Facebook appealed to far-right groups and how these features influenced users who incorporated far-right themes and narratives, Hutchinson and Droogan (2022) attempted to find these answers by conducting a cross-national comparative analysis over three years (2016–2019) using data from 59 Australian and Canadian far-right extremist groups on Facebook. Here, the level of user engagement with the posts was assessed using “Facebook reactions” and identified the themes and narrative and generated user engagements, which mostly comprised six ‘“Facebook reactions.” In-depth, the qualitative analysis of the narrative and themes show that the highest user engagement is found with “anger” and “love” reactions paired together. These two reactions were the most frequently used, particularly in narratives and themes relating to in-group and out-group dynamics. Additionally, algorithms further amplified the visibility and impact of these reactions within these groups (Hutchinson and Droogan, 2023).

Inciting public anger, especially raising controversial issues, is a rewarding tactic to increase motivation and contribute to high-threshold interaction. Gerbaudo et al. (2023) called it “anger-triggering communication.” They state, “[t]he right-wing populists have a significantly higher number of ‘Angry’ Facebook reactions per post compared to their political adversaries; there is a positive and significant effect of the number of Angry reactions on the number of times a post is shared; Angry reactions and Shares are overrepresented in posts on immigration and security, but anger-fueled mobilization is not limited to these topics” (Gerbaudo et al., 2023). Their findings contribute to the understanding of emotional communication, populism, the effectiveness of negative campaigning, and its mobilization.

#### 3.2.4 “Facebook reactions” in news and junk news

In a particular topic such as mis/disinformation, how do users make sense to interact with political junk news on Facebook, How is the “emotional architecture” constructed to intervene in this sense-building or interactive process? These questions were addressed through topic modeling of 40,500 junk news articles. Savolainen et al. (2020) explore the interplay between junk news, the audience, and Facebook’s emotional architecture, generating bivalent emotional logic. “Love” and “angry” rarely co-occurred and strongly correlated with other reactions. Savolainen et al. (2020) contend that the “anger” reaction seems to co-occur predominantly with the “sad” and “wow” reactions, whereas the relation between “like” and “love” reactions contains high positivity. This division of Facebook posts between positive and negative carried out further arguments for the characteristics of junk news engagement.

Another research was carried out by Sturm Wilkerson et al. (2021) about the interaction between the “hyperpartisan” news content on Facebook and audience reactions in the US in 2016. The study combined political communication, social media, affective intelligence, and emotional symbolism. It then, examined both the messages on political-leaning Facebook pages and the audience responses to hyperpartisan content. The research question addressed the content of hyperpartisan posts on Facebook, with the relationships between content and users’ engagement. The authors found that social media messages contained more “anger” reactions for audiences of both right- and left-leaning pages (Sturm Wilkerson et al., 2021), and news generating negative reactions were circulated and shared more than those generating positive reactions. The study found the emotional appeals for the prediction of “Facebook reactions.” Basile et al. (2018b) presented a study based on the “Facebook reactions” feature to predict the entropy of a post’s reactions; the reactions were further measured as a proxy to predict the controversy of news, resulting in high entropy (high reactions) with bigger controversy.

During the Mexican elections in 2018, de León and Trilling (2021) conducted a study focusing on positive versus negative political content related to emotional responses, and the degree of influence of the articles that were shared across social media in the context of that election. They found a strong relationship between the “sad” reaction and negative news and between the “wow” reaction and negative news. The last finding shows that the “wow” reaction is used as a negative expression (i.e., disbelief rather than amazement) when it comes to political news. They also found a bias in news-sharing behavior and engagement with a great amount of “anger” reaction to political news marked with violence and corruption.

In a significant case of the former British colony Hong Kong, where a political movement was ongoing after the National Security Law was passed, Nip and Berthelier (2023) conducted a study to understand the impact of cultural disparities on emotional responses to political news. They found that China-critical news pages expressed the highest emotional intensity (expressed the most anger); however, their news-sharing correlates with “wow” and “sad” reactions. While readers of China’s media in Hong Kong had expressed the lowest emotional intensity (“love”), China-supporting media readers fell in between them.

Savolainen et al. (2020) argued and discovered that the interplay between junk news (producers), the audience, and Facebook’s emotional architecture generates a bivalent emotional logic: “love” and “anger” reactions rarely co-occurred and correlated most strongly with other reactions.

### 3.3 Other social issues (sub-topics: psychological, sentimental and emotional, health care, business, and customer engagement, miscellaneous)

In social media, emojis are used widely and are commonly assumed to express the emotional state of the user (Tian et al., 2017). Since February 2016, Facebook users have been able to express their emotions in response to the newly introduced “Facebook reactions” feature (Pool and Nissim, 2016), which allows users to express their psychological emotions and feelings regarding published content (Raad et al., 2018).

This feature of Facebook helps to better understand the users and collect information on their preferences and likeness to be used in different areas such as business and advertisements (Hutchinson, 2016; Baldwin, 2021) or healthcare (Rovetta, 2022).

Tian et al. (2017) consider that social media is not only for sharing information but for the expression of emotion. Consequently, they argued over the upscaled size, genres, and languages or cultures dataset for investigating the types of emojis used in different emotional contexts. They found that “Facebook reactions” are a good data source for investigating the indicators of users’ emotional attitudes. Reliable “Facebook reactions” such as “anger” and “love” indicate overall sentiment. Moreover, there is a correlation between Facebook’s reactions and emoji usage. Thus, emojis can be used to detect users’ sentiments too. Giuntini et al. (2019) made it possible to determine the polarity (positive, negative, and neutral) of “Facebook reactions” and their correlation with emotional expressions; they suggested that the use of reactions could increase confidence in determining the emotions for the contents and emotional reflection of users. If we consider the collective attitude and performance through image circulation on Facebook, the reaction button makes it possible to study them. Geboers et al. (2020) used a relational approach to determine photos that have had audiences and been contextualized with mixed reactions, often showing a correlation between “sad” and “anger” as well as “love” and “sad.” Pool and Nissim (2016) conducted a study before to explore the potential of using Facebook reactions to perform emotion classification. They were finally able to develop a single model for each emotion classification. A similar approach was made by Raad et al. (2018) to create a framework for predicting the reaction distribution on Facebook posts. They examined the potential Facebook reaction usage, recognized classified emotions, and found the result through their dataset, which revealed that “Like,” “haha,” and “love,” were the most frequently used “Facebook reactions.” Graziani et al. (2019) proposed the task of emotion classification and prediction of “Facebook reactions” and warned that Facebook reactions could easily become ambiguous in mapping emotion classes. Later, Wisniewski et al. (2020) identified many design constraints of the “Facebook reaction” feature and users’ inability to express conflicting emotions.

Using Big Five personality and narcissism theories, aimed to investigate how users used “Facebook reactions,” over which content, and what personality factors motivated them to use. They found that the audience used more positive than negative reactions, concerning the established online norms and with positive self-presentation. They put reactions on status and updates, images, and at least for events. Furthermore, “Reaction use is predicted by high neuroticism, extraversion, and openness, and low narcissism, with these factors driving the use of the strong love reaction, the negative sad and angry reactions, and on the specifically personal time-hop content, possibly motivated by a desire for complex social interaction and to positively manage online interactions, and on the and pictorially salient picture and link content types”. If we consider emotional content, according to Paletz et al. (2023), emotions are associated with posts either positive or negative or both, and relatively lower and higher degrees of activation/arousal emotions, even if they control the number of followers and “Facebook reactions.”

## 4 Conclusion

This study suggests that “Facebook reactions” have been extensively studied as specific indicators of engagement across various broad topics. We found the basic five Facebook reactions with their nuanced patterns of reaction distribution based on specific research areas and topics.

Our analysis suggests that “love” and “haha” Facebook reactions are always considered positive responses; however, in certain cases, the “haha” and “wow” reactions can be a negative engagement indicator with ambiguous meanings. “Sad” and “anger” are always negative indicators of interactions.

Individual articles can draw different conclusions; although in the major combined topics, we have found a unique similarity, especially for Politics and Far-right groups, where emotion plays an important role in user engagement with Facebook reactions and content. A particular pattern of behavior observed in the studies under analysis is that positive emotions usually influence positive comments with positive reactions, while negative emotions influence negative comments with negative reactions on Facebook. The negative sentiment with user engagement demonstrates that negativity is the consistent factor for user connections on Facebook, and more negative reactions generate higher user engagement. It is interesting that political communication triggers emotional reactions and influences the frequency of post-sharing with sadness and angry reactions. Thus, these populist communication strategies increase the outreach of their messages and stimulate users’ interactions with Facebook reactions. In addition, a relationship exists between specific emotions such as fear, anger, enthusiasm, and pride with the engagement of users.

As social media expands, users increasingly interact with different topics and their areas of interest with different current challenges. The researchers frequently used different data sources and collection methods. Combined with the complexities of the subject, the absence of a unified source of Facebook data may have contributed to some conflicting results. The matter of concern was not only a unified data source but also the overlapping of research topics, whereas Facebook reactions played an intermediary role in determining various degrees and intensity of the user’s psychological and emotional levels with many contrasts and other factors of sociocultural norms, which always differed into certain geographical areas and their cultures. Thus, the outcome of those studies always seemed to differ.

An increasing number of studies were found with quantitative data, which were obtained by Facebook, although they were neither up to date nor relevant but for a specific time duration. Those studies have provided insights into users’ behavior and Facebook reactions dealing with wide areas and different topics. However, event-based tabloid topics, including those concerning sensational or scandalous subjects, were always ignored.

We were expecting Cultural Studies and Cross-Cultural Analysis of Facebook reactions, either as a comparative or interpretative study across the different cultural contexts. The versatility of Facebook reactions could be used for studying a wide range of fields with the integration of interdisciplinary collaboration and other studies. Researchers could examine the patterns of Facebook reactions within online communities and subcultures, such as niche interest groups, hobbyist communities, or fan groups. From this perspective, the availability of advanced large language models that can categorize large volumes of social media content more effectively and with less resource expenditure opens intriguing possibilities for analyzing how reactions to posts vary across different topics.

The future of Facebook’s reaction is likely to be multifaceted, overlapping a wide range of disciplines, and integrating with other data sources and research methodologies. We hope upcoming research will continue to refine the approaches in the exciting area of Facebook reactions and user engagement with Misinformation and Fact-Checking, Digital-Activism and Social Movements, and so on.

## Data availability statement

The datasets presented in this study can be found in online repositories. The names of the repository/repositories and accession number(s) can be found in the article/Supplementary material.

## Author contributions

SA: Writing – original draft, Writing – review & editing. FG: Supervision, Writing – review & editing.
