# Supplementary material for: Facebook reactions in the context of politics and social issues: a systematic literature review
Source: Front Sociol. 2024 May 15;9:1379265. doi: 10.3389/fsoc.2024.1379265 (PMC11135286; doi:10.3389/fsoc.2024.1379265)
Supplement: Supplementary file 1 [file Data_Sheet_1.docx]

Supplementary Material

# The following supplementary tables catalog the entire dataset of 64 studies considered in this analysis. The dataset is divided into several categories as follows: Table 1: Introduction of Facebook Reactions (N=6), Table 2: Politics and Far-Right Groups Related Articles (N=19), Table 3a: Other Social Issues (Business and Customer Engagement) (N=6), Table 3b: Other Social Issues (Psychological, Sentimental, and Emotional) (N=17), Table 3c: Other Social Issues (Healthcare) (N=8), and Table 3d: Other Social Issues (Miscellaneous) (N=8). When available, a brief commentary is provided in the "Result and Observation" column.

# Supplementary Tables

| Articles | Result and Observation |
| --- | --- |
| Turnbull, S., & Jenkins, S. (2016). Why Facebook Reactions are good news for evaluating social media campaigns. *Journal of Direct, Data and Digital Marketing Practice*, *17*(3), 156–158. https://doi.org/10.1057/dddmp.2015.56 | An opportunity for marketers to measure such emotional engagement. |
| Varanasi, R. A., Dicicco, E., & Gambino, A. (2018). Facebook Reactions: Impact of Introducing New Features of SNS on Social Capital. In C. Stephanidis (Ed.), *HCI International 2018 – Posters’ Extended Abstracts* (Vol. 850, pp. 444–451). Springer International Publishing. https://doi.org/10.1007/978-3-319-92270-6_64 | Help us understand the relationship maintenance, group cohesion, |
| ADDIN ZOTERO_BIBL {"uncited":[],"omitted":[],"custom":[]} CSL_BIBLIOGRAPHY Smoliarova, A., Gromova, T., & Pavlushkina, N. (2018). Emotional Stimuli in Social Media User Behavior: Emoji Reactions on a News Media Facebook Page. *Internet Science*, 242–256. https://doi.org/10.1007/978-3-030-01437-7_19 | Love icon is used in the most mono-semantic way; Posts evoking a Sad reaction are less likely to be commented on or shared than posts arousing any other reaction |
| Wisniewski, P., Badillo-Urquiola, K., Ashtorab, Z., & Vitak, J. (2020). Happiness and Fear: Using Emotions as a Lens to Disentangle How Users Felt About the Launch of Facebook Reactions. *ACM Transactions on Social Computing*, *3*(4), 20:1-20:25. https://doi.org/10.1145/3414825 | After launch users were more positive about the feature; users’ inability to express conflicting emotions. |
| Merrill, J. B., & Oremus, will. (2021, October 26). *Five points for anger, one for a ‘like’: How Facebook’s formula fostered rage and misinformation*. Washington Post. https://www.washingtonpost.com/technology/2021/10/26/facebook-angry-emoji-algorithm/ |  |
| Paolillo, J. C. (2023). The awkward semantics of Facebook reactions. *First Monday*. https://doi.org/10.5210/fm.v28i8.13157 | The reactions’ distribution is complex and unstable across samples |

# Table - 1) Introduction of Facebook Reactions

| Articles | Result and Observation |
| --- | --- |
| Turnbull, S., & Jenkins, S. (2016). Why Facebook Reactions are good news for evaluating social media campaigns. *Journal of Direct, Data and Digital Marketing Practice*, *17*(3), 156–158. https://doi.org/10.1057/dddmp.2015.56 | An opportunity for marketers to measure such emotional engagement. |
| Larsson, A. (2017). Diversifying Likes—Relating Reactions to Commenting and Sharing on Newspaper Facebook Pages. *Journalism Practice*. https://doi.org/10.1080/17512786.2017.1285244 | More positive forms of Reactions appear to have a hampering effect on the willingness of news consumers; more negative varieties of Facebook Reactions appear to yield adverse influences. |
| Basile, A., Caselli, T., Merenda, F., & Nissim, M. (2018). Facebook Reactions as Controversy Proxies: Predictive Models over Italian News. *Italian Journal of Computational Linguistics*, *4*(2), 73–89. https://doi.org/10.4000/ijcol.514 | Controversy and reactions can be modeled successfully at various degrees of granularity. |
| Basile, A., Caselli, T., & Nissim, M. (2018). Predicting Controversial News Using Facebook Reactions. In R. Basili & G. Satta (Eds.), *Proceedings of the Fourth Italian Conference on Computational Linguistics CLiC-it 2017: 11-12 December 2017, Rome* (pp. 12–17). Accademia University Press. https://doi.org/10.4000/books.aaccademia.2370 | Results are promising, given that the model beats the baseline in almost all cases in cross-validation of the same source data. |
| Keller, T. R., & Kleinen-von Königslöw, K. (2018). Followers, Spread the Message! Predicting the Success of Swiss Politicians on Facebook and Twitter. *Social Media + Society*, *4*(1), 2056305118765733. https://doi.org/10.1177/2056305118765733 | Digital followership in turn influences the average number of digital reactions on Facebook. |
| Mancosu, M. (2018). Populism, Emotionalized Blame Attribution and Selective Exposure in Social Media. A Comparative Analysis of Italy and UK. *Comunicazione Politica*, *1*, 73–92. https://doi.org/10.3270/89738 | Allows users to indicate the emotions that a post provokes in them (sadness, joy, anger, etc.). |
| Sandoval-Almazan, R., & Valle-Cruz, D. (2018). Facebook impact and sentiment analysis on political campaigns. *Proceedings of the 19th Annual International Conference on Digital Government Research: Governance in the Data Age*, 1–7. https://doi.org/10.1145/3209281.3209328 | Facebook emoticons gave some approach to the perception of voter’s sympathy or interest in the candidate. |
| Al-Rawi, A. (2019). Networked emotional news on social media. *Networked Emotional News on Social Media, Journalism Practice*, *14*(9), 1125–1141. https://doi.org/10.1080/17512786.2019.1685902 | Readers are emotionally engaged with news that involves positive feelings, especially love. |
| Blassnig, S., & Wirz, D. S. (2019). Populist and Popular: An Experiment on the Drivers of User Reactions to Populist Posts on Facebook. *Social Media + Society*, *5*(4), 2056305119890062. https://doi.org/10.1177/2056305119890062 | Users with strong populist attitudes share populist messages more often than they share non-populist messages. |
| Burger, P., Kanhai, S., Pleijter, A., & Verberne, S. (2019). The reach of commercially motivated junk news on Facebook. *PLOS ONE*, *14*(8), e0220446. https://doi.org/10.1371/journal.pone.0220446 | Junk news pages have been increasingly successful in attracting user engagement. |
| Gerbaudo, P., Marogna, F., & Alzetta, C. (2019). When “Positive Posting” Attracts Voters: User Engagement and Emotions in the 2017 UK Election Campaign on Facebook. *Social Media + Society*, *5*(4), 2056305119881695. https://doi.org/10.1177/2056305119881695 | Need for a more balanced understanding of the relationship between content, emotions, and user engagement. |
| Eberl, J.-M., Tolochko, P., Jost, P., Heidenreich, T., & Boomgaarden, H. (2020). What’s in a post? How sentiment and issue salience affect users’ emotional reactions on Facebook. *Journal of Information Technology & Politics*, *17*. https://doi.org/10.1080/19331681.2019.1710318 | Effect of sentiment on “Angry” Reactions is highest. |
| Jost, P., Maurer, M., & Hassler, J. (2020). Populism Fuels Love and Anger: The Impact of Message Features on Users’ Reactions on Facebook. *International Journal of Communication*, *14*(0), Article 0. https://doi.org/1932–8036/20200005 | Love and Angry can be categorized as positive and negative one-click expressions of emotional states. |
| Sandoval-Almazan, R., & Valle-Cruz, D. (2020). Sentiment Analysis of Facebook Users Reacting to Political Campaign Posts. *Digital Government: Research and Practice*, *1*(2), 1–13. https://doi.org/10.1145/3382735 | The winning political party had more negative sentiment. |
| Savolainen, L., Trilling, D., & Liotsiou, D. (2020). Delighting and Detesting Engagement: Emotional Politics of Junk News. *Social Media + Society*, *6*(4), 2056305120972037. https://doi.org/10.1177/2056305120972037 | Junk news brings otherwise disparate audience members together and orients their dramatic focus toward objects of collective joy, anger, or concern. |
| de León, E., & Trilling, D. (2021). A Sadness Bias in Political News Sharing? The Role of Discrete Emotions in the Engagement and Dissemination of Political News on Facebook. *Social Media + Society*, *7*(4), 20563051211059710. https://doi.org/10.1177/20563051211059710 | A negativity bias in news sharing and engagement, showing an outsized prevalence of anger in response to political news. |
| Muraoka, T., Montgomery, J., Lucas, C., & Tavits, M. (2021). Love and Anger in Global Party Politics: Facebook Reactions to Political Party Posts in 79 Democracies. *Journal of Quantitative Description: Digital Media*, *1*, 1–38. https://doi.org/10.51685/jqd.2021.005 | Parties receive systematically different proportions of Love and Angry reactions depending on their ideology, party family, and populist orientation. |
| Sturm Wilkerson, H., Riedl, M. J., & Whipple, K. N. (2021). Affective Affordances: Exploring Facebook Reactions as Emotional Responses to Hyperpartisan Political News. *Digital Journalism*, *9*(8), 1040–1061. https://doi.org/10.1080/21670811.2021.1899011 | Analyze emotional reactions elicited through Facebook Reactions in response to right- and left-leaning Facebook news posts. |
| Zerback, T., & Wirz, D. S. (2021). Appraisal patterns as predictors of emotional expressions and shares on political social networking sites. *Studies in Communication Sciences*, *21*(1), Article 1. https://doi.org/10.24434/j.scoms.2021.01.003 | If posts that include sadness or anger are associated with the corresponding emotional reactions in the form of emojis, these posts are shared more often. |
| Bagić Babac, M. (2022). Emotion analysis of user reactions to online news. *Information Discovery and Delivery*, *ahead-of-print*(ahead-of-print). https://doi.org/10.1108/IDD-04-2022-0027 | Comprehensive understanding of the engagement could be a better predictor of future behavior. |
| Bil-Jaruzelska, A., & Monzer, C. (2022). All About Feelings? Emotional Appeals as Drivers of User Engagement With Facebook Posts. *Politics and Governance*, *10*(1), 172–184. https://doi.org/10.17645/pag.v10i1.4758 | Engagement with a post substantially increases when appeals to anger, enthusiasm, and pride are present; no relationship between appeals to fear and engagement. |
| Sandberg, L., Jacobs, K., & Spierings, N. (2022). Populist MPs on Facebook: Adoption and emotional reactions in Austria, the Netherlands, and Sweden. *Scandinavian Political Studies*, *45*(4), 504–528. https://doi.org/10.1111/1467-9477.12239 | Facebook posts of populist MPs activate feelings of indignation, triggering more emotional responses; “haha” also reflects the sarcastic ridiculing of political opponents, paving the way for anger. |
| Klinger, U., Koc-Michalska, K., & Russmann, U. (2023). Are Campaigns Getting Uglier, and Who Is to Blame? Negativity, Dramatization and Populism on Facebook in the 2014 and 2019 EP Election Campaigns. *Political Communication*, *40*(3), 263–282. https://doi.org/10.1080/10584609.2022.2133198 | Populist content also led to more user reactions, Negative, exaggerated, and sensationalized messaging therefore makes sense from a strategic perspective. |
| Kluknavská, A., Novotná, M., & Eisele, O. (2023). Fuming Mad and Jumping with Joy: Emotional Responses to Uncivil and Post-Truth Communication by Populist and Non-Populist Politicians on Facebook During the COVID-19 Crisis. *Mass Communication and Society*, *0*(0), 1–25. https://doi.org/10.1080/15205436.2023.2252396 | Uncivil and post-truth message elements, affiliation with a populist party, and pandemic Influenced the volume of emotional interactions with political posts. |
| Macdonald, M., Russell, A., & Hua, W. (2023). Negative Sentiment and Congressional Cue-Taking on Social Media. *PS: Political Science & Politics*, *56*(2), 201–206. https://doi.org/10.1017/S1049096522001299 | New evidence demonstrating the power of that negative sentiment to elicit more user engagement. |
| Nip, J. Y. M., & Berthelier, B. (2023). Emotional Profiles of Facebook Pages: Audience Response to Political News in Hong Kong. *Journalism and Media*, *4*(4), Article 4. https://doi.org/10.3390/journalmedia4040065 | The emotion most associated with their political news sharing. |
| Pérez-Seoane, J., Manuel Corbacho-Valencia, J., & Dafonte-Gómez, A. (2023). An analysis of the most viral posts from Ibero-American fact-checkers on Facebook in 2021. *Revista ICONO 14. Revista Científica de Comunicación y Tecnologías Emergentes*, *21*(1), 1–20. https://doi.org/10.7195/ri14.v21i1.1951 | Interactions stem from emotional reactions, predominantly those associated with positive feelings. |
| Matamoros Fernandez, A. (2018). Inciting anger through Facebook reactions in Belgium: The use of emoji and related vernacular expressions in racist discourse. *First Monday*, *23*(9), Article 9. | Represent an opportunity to investigate the material politics of platforms and explore their role in racist discourse. |
| Tasente, T., & Rus, M. (2019). Donald Trump’s Social Media Communication or the voice of a man is stronger than the voice of an institution. *Technium Social Sciences Journal*, *1*, 1–8. https://doi.org/10.47577/tssj.v1i1.27 | Identified and analyzed the messages that generate high engagement from users; and the dominant reactions generated by the online audience. |
| Hutchinson, J., & Droogan, J. (2022). Far-right “Reactions”: A comparison of Australian and Canadian far-right extremist groups on Facebook. *Behavioral Sciences of Terrorism and Political Aggression*. https://doi.org/10.1080/19434472.2022.2112742 | Interpretation of far-right extremist themes and narrative and user engagement. |
| Gerbaudo, P., De Falco, C. C., Giorgi, G., Keeling, S., Murolo, A., & Nunziata, F. (2023). Angry Posts Mobilize: Emotional Communication and Online Mobilization in the Facebook Pages of Western European Right-Wing Populist Leaders. *Social Media + Society*, *9*(1), 20563051231163327. https://doi.org/10.1177/20563051231163327 | Highlights that stoking public anger, especially around controversial issues such as immigration and security. |
| Hutchinson, J., & Droogan, J. (2023, January 30). More Than Just Pretty Pictures: A Comparison of Australian and Canadian Far-Right Extremist ‘Reaction’ Usage on Facebook. *GNET*. https://gnet-research.org/2023/01/30/more-than-just-pretty-pictures-a-comparison-of-australian-and-canadian-far-right-extremist-reaction-usage-on-facebook/ | User’s interpretation of extremist ideology. |
| Oliveira, L., & Azevedo, J. (2023). Using Social Media Categorical Reactions as a Gateway to Identify Hate Speech in COVID-19 News. *Sn Computer Science*, *4*(1), 11. https://doi.org/10.1007/s42979-022-01421-5 | Negative emotions alone do not always indicate the presence of hate speech. |

# Table - 2) Politics and Far-right groups related Articles.

| Articles |
| --- |
| Krebs, F., Lubascher, B., Moers, T., Schaap, P., & Spanakis, G. (2017). *Social Emotion Mining Techniques for Facebook Posts Reaction Prediction* (arXiv:1712.03249). arXiv. https://doi.org/10.48550/arXiv.1712.03249 |
| Antoniadis, I., Paltsoglou, S., & Patoulidis, V. (2019). Post popularity and reactions in retail brand pages on Facebook. *International Journal of Retail & Distribution Management*, *47*(9), 957–973. https://doi.org/10.1108/IJRDM-09-2018-0195 |
| Lee, Y.-I., Phua, J., & Wu, T.-Y. (2020). Marketing a health Brand on Facebook: Effects of reaction icons and user comments on brand attitude, trust, purchase intention, and eWOM intention. *Health Marketing Quarterly*, *37*(2), 138–154. https://doi.org/10.1080/07359683.2020.1754049 |
| Yang, M., Ren, Y., & Adomavicius, G. (2020). Engagement by Design: An Empirical Study of the “Reactions” Feature on Facebook Business Pages. *ACM Transactions on Computer-Human Interaction*, *27*(6), 1–35. https://doi.org/10.1145/3412844 |
| Dehouche, N. (2020). Dataset on usage and engagement patterns for Facebook Live sellers in Thailand. *Data in Brief*, *30*, 105661. https://doi.org/10.1016/j.dib.2020.105661 |
| Vaiciukynaite, E., Zickute, I., & Salkevicius, J. (2022). *Solutions of Brand Posts on Facebook to Increase Customer Engagement Using the Random Forest Prediction Model* (pp. 191–214). https://doi.org/10.1007/978-3-031-11371-0_9 |

# Table – 3a) Other Social Issues (Business and customer engagement)

| Articles |
| --- |
| Pool, C., & Nissim, M. (2016). *Distant supervision for emotion detection using Facebook reactions* (arXiv:1611.02988). arXiv. https://doi.org/10.48550/arXiv.1611.02988 |
| Tian, Y., Galery, T., Dulcinati, G., Molimpakis, E., & Sun, C. (2017). Facebook sentiment: Reactions and Emojis. *Proceedings of the Fifth International Workshop on Natural Language Processing for Social Media*, 11–16. https://doi.org/10.18653/v1/W17-1102 |
| Kuo, P. C., Alvarado, F. H. C., & Chen, Y.-S. (2018). *Facebook Reaction-Based Emotion Classifier as Cue for Sarcasm Detection* (arXiv:1805.06510). arXiv. https://doi.org/10.48550/arXiv.1805.06510 |
| Raad, B. T., Philipp, B., Patrick, H., & Christoph, M. (2018). ASEDS: Towards Automatic Social Emotion Detection System Using Facebook Reactions. *2018 IEEE 20th International Conference on High Performance Computing and Communications; IEEE 16th International Conference on Smart City; IEEE 4th International Conference on Data Science and Systems (HPCC/SmartCity/DSS)*, 860–866. https://doi.org/10.1109/HPCC/SmartCity/DSS.2018.00143 |
| Giuntini, F. T., Ruiz, L. P., Kirchner, L. D. F., Passarelli, D. A., Dos Reis, M. D. J. D., Campbell, A. T., & Ueyama, J. (2019). How Do I Feel? Identifying Emotional Expressions on Facebook Reactions Using Clustering Mechanism. *IEEE Access*, *7*, 53909–53921. https://doi.org/10.1109/ACCESS.2019.2913136 |
| Freeman, C., Roy, M. K., Fattoruso, M., & Alhoori, H. (2019). Shared Feelings: Understanding Facebook Reactions to Scholarly Articles. *2019 ACM/IEEE Joint Conference on Digital Libraries (JCDL)*, 301–304. https://doi.org/10.1109/JCDL.2019.00050 |
| Rahman, M. A., & Seddiqui, M. H. (2019). *Comparison of Classical Machine Learning Approaches on Bangla Textual Emotion Analysis* (arXiv:1907.07826). arXiv. https://doi.org/10.48550/arXiv.1907.07826 |
| Graziani, L., Melacci, S., & Gori, M. (2019). *Jointly Learning to Detect Emotions and Predict Facebook Reactions* (arXiv:1909.10779). arXiv. https://doi.org/10.48550/arXiv.1909.10779 |
| Freeman, C., Alhoori, H., & Shahzad, M. (2020). Measuring the Diversity of Facebook Reactions to Research. *Proceedings of the ACM on Human-Computer Interaction*, *4*(GROUP), 1–17. https://doi.org/10.1145/3375192 |
| Geboers, M., Stolero, N., Scuttari, A., Vliet, L. V., & Ridley, A. (2020). Why Buttons Matter: Repurposing Facebook’s Reactions for Analysis of the Social Visual. *International Journal of Communication*, *14*(2020), Article 0. |
| Wisniewski, P., Badillo-Urquiola, K., Ashtorab, Z., & Vitak, J. (2020). Happiness and Fear: Using Emotions as a Lens to Disentangle How Users Felt About the Launch of Facebook Reactions. *ACM Transactions on Social Computing*, *3*(4), 20:1-20:25. https://doi.org/10.1145/3414825 |
| Scott, G. G., Conlon, L. J., & Wilson, C. (2020). Facebook Reactions: How are They Used and Which Personality Factors Predict their Use? *The Journal of Social Media in Society*, *9*(2), Article 2. |
| Jayawickrama, V., Weeraprameshwara, G., de Silva, N., & Wijeratne, Y. (2022). Facebook for Sentiment Analysis: Baseline Models to Predict Facebook Reactions of Sinhala Posts. *The International Journal on Advances in ICT for Emerging Regions*, *15*(2), 22–32. |
| Pratama, A. (2022). Sentiment Analysis of Facebook Posts through Special Reactions: The Case of Learning from Home in Indonesia Amid COVID-19. *Jurnal Ilmiah Teknik Elektro Komputer Dan Informatika (JITEKI)*, *8*(1), 83–92. https://doi.org/10.26555/jiteki.v8i1.23615 |
| Chin, C.-Y., & Huang, W.-Y. (2023). Discovering fans and anti-fans among social media users based on their emotional reactions and comments. *Journal of Information Science*, 01655515231162284. https://doi.org/10.1177/01655515231162284 |
| Shahzad, M., Freeman, C., Rahimi, M., & Alhoori, H. (2023). Predicting Facebook sentiments towards research. *Natural Language Processing Journal*, *3*, 100010. https://doi.org/10.1016/j.nlp.2023.100010 |
| Paletz, S. B. F., Johns, M. A., Murauskaite, E. E., Golonka, E. M., Pandža, N. B., Rytting, C. A., Buntain, C., & Ellis, D. (2023). Emotional content and sharing on Facebook: A theory cage match. *Science Advances*, *9*(39), eade9231. https://doi.org/10.1126/sciadv.ade9231 |

# Table – 3b) Other Social Issues (Psychological, Sentimental and Emotional)

| Articles |
| --- |
| Tran, T., Nguyen, D., Nguyen, A., & Golen, E. (2018). Sentiment analysis of marijuana content via Facebook emoji-based reactions. *2018 IEEE International Conference on Communications (ICC)*, 1–6. https://doi.org/978-1-5386-3180-5/18 |
| Balakrishnan, V., Govindan, V., Arshad, N. I., Shuib, L., & Cachia, E. (2019). Facebook User Reactions and Emotion: An Analysis of Their Relationships among the Online Diabetes Community. *Malaysian Journal of Computer Science*, *Special Issue 2019*(Industrial Revolution: Impact and Readiness Special Issue, 2019), 87–97. https://doi.org/10.22452/mjcs.sp2019no3.6 |
| Pócs, D., Adamovits, O., Watti, J., Kovács, R., & Kelemen, O. (2021). Facebook Users’ Interactions, Organic Reach, and Engagement in a Smoking Cessation Intervention: Content Analysis. *Journal of Medical Internet Research*, *23*(6), e27853. https://doi.org/10.2196/27853 |
| Hrincu, V., An, Z., Joseph, K. A., Jiang, Y. F., Shi, E., & Robillard, J. M. (2022). Dementia Research Engagement on Social Media: A Content Analysis. *Alzheimer’s & Dementia*, *18*(S8), e060826. https://doi.org/10.1002/alz.060826 |
| Rovetta, A. (2022). World Health Organization is losing online credibility towards health-sensitive topics: Infodemiological analysis of Facebook users’ reactions. *Health Promotion Perspectives*, *12*(4), 367–371. https://doi.org/10.34172/hpp.2022.48 |
| Kilgo, D. K., & Midberry, J. (2022). Social Media News Production, Emotional Facebook Reactions, and the Politicization of Drug Addiction. *Health Communication*, *37*(3), 375–383. https://doi.org/10.1080/10410236.2020.1846265 |
| Watti, J., Millner, M., Siklósi, K., Kiss, H., Kelemen, O., & Pócs, D. (2022). Smokers’ Engagement Behavior on Facebook: Verbalizing and Visual Expressing the Smoking Cessation Process. *International Journal of Environmental Research and Public Health*, *19*(16), 9983. https://doi.org/10.3390/ijerph19169983 |
| Yavetz, G., & Aharony, N. (2023). Information under lockdown: A content analysis of government communication strategies on Facebook during the COVID-19 outbreak. *Heliyon*, *9*(4), e15562. https://doi.org/10.1016/j.heliyon.2023.e15562 |

# Table – 3c) Other Social Issues (HealthCare)

| Articles |
| --- |
| Ross, B., Potthoff, T., Majchrzak, T. A., Chakraborty, N. R., Ben Lazreg, M., & Stieglitz, S. (2018, January 3). *The Diffusion of Crisis-Related Communication on Social Media: An Empirical Analysis of Facebook Reactions*. 51st Hawaii International Conference on System Sciences \|. http://hdl.handle.net/10125/50207 |
| Oliveira, L., & Azevedo, J. (2021). Profiling Media Outlets and Audiences on Facebook: COVID-19 Coverage, Emotions and Controversy. *Proceedings of the 17th International Conference on Web Information Systems and Technologies*, 186–196. https://doi.org/10.5220/0010717400003058 |
| Masullo, G. M. (2022). Facebook reactions as heuristics: Exploring relationships between reactions and commenting frequency on news about COVID-19. *First Monday*. |
| Al-Zaman, Md. S., & Ahona, T. (2022). Users’ Reactions to Rape News Shared on Social Media: An Analysis of Five Facebook Reaction Buttons. *Asian Journal for Public Opinion Research*, *10*, 51–73. https://doi.org/10.15206/ajpor.2022.10.1.51 |
| Cantrell, S. J., Winters, R. M., Kaini, P., & Walker, B. N. (2022). Sonification of Emotion in Social Media: Affect and Accessibility in Facebook Reactions. *Proceedings of the ACM on Human-Computer Interaction*, *6*(CSCW1), 1–26. https://doi.org/10.1145/3512966 |
| Johnson, C. (2023). *Courts Are Going to “Love” This: An Analysis of the Facebook “Reactions” as Hearsay* (SSRN Scholarly Paper 4364581). https://doi.org/10.2139/ssrn.4364581 |
| Etta, G., Sangiorgio, E., Marco, N. D., Avalle, M., Scala, A., Cinelli, M., & Quattrociocchi, W. (2023). Characterizing engagement dynamics across topics on Facebook. *PLOS ONE*, *18*(6), e0286150. https://doi.org/10.1371/journal.pone.0286150 |
| Cui, Y., Fang, Z., & Wang, X. (2023). Article promotion on Twitter and Facebook: A case study of Cell journal. *Journal of Information Science*, *49*(5), 1218–1228. https://doi.org/10.1177/01655515211059772 |

# Table – 3d) Other Social Issues (Miscellaneous)
